# Editorial: The Role of the Immune Response in Brain Metastasis

**DOI:** 10.3389/fonc.2022.922700

**Published:** 2022-05-10

**Authors:** Nicola R. Sibson, Frits Thorsen, Manuel Sarmiento Soto

**Affiliations:** ^1^ Department of Oncology, University of Oxford, Oxford, United Kingdom; ^2^ Molecular Imaging Center, Department of Biomedicine, University of Bergen, Bergen, Norway; ^3^ Department of Biochemistry and Molecular Biology, University of Seville, Seville, Spain; ^4^ Instituto de Biomedicina de Sevilla, Hospital Universitario Virgen del Rocío/CSIC/ Universidad de Sevilla and CIBERNED, Seville, Spain

**Keywords:** brain metastasis, immune response, tumour microenvironment, immunotherapy, imaging

Conventionally, metastases are treated using combinations of surgery, radiotherapy and systemic therapy. Nevertheless, survival amongst patients suffering metastatic spread to the brain remains extremely poor. Treatment failure frequently reflects the impact of complex and variable factors within the unique brain microenvironment, resulting in resistance to therapy and the onset of an immunosuppressive tumour microenvironment (TME). Thus, the development of new therapeutic strategies targeting brain-specific TME components has become one of the biggest challenges in the field.

It is becoming clear that the immune response to brain metastasis evolves over the time-course of tumour progression, and can have both tumour suppressive and tumour promoting effects. Nevertheless, as demonstrated by both Hu et al. and Xiao et al., despite issues of access across the blood-brain barrier, immunotherapy can confer a survival benefit in cancer patients with brain metastases. The overarching goal of this Research Topic, therefore, was to discuss the current state of knowledge with regards to the immune response to brain metastasis, and its potential role as a target for both diagnosis and treatment. From the available volumes on the Research Topic, we collected both original papers and review articles that addressed individual components of the brain immune response and TME, novel immunotherapeutic approaches and targeting of immune biomarkers for improved diagnosis or treatment planning.

It is undoubtedly the case that the complexity of the brain microenvironment contributes to the challenges encountered in treating brain metastases. Moreover, in the presence of a tumour, interactions between both the systemic and central immune responses and the normal brain environment only serve to compound these challenges. Understanding changes in the brain/tumour microenvironment at the cellular and molecular level is key to the development of new therapeutic strategies. In their primary research article, Economopoulos et al. evaluate the contribution of both blood-derived and brain resident macrophages (microglia) to brain metastasis development, with particular reference to their pro-/anti-inflammatory state. The results of this study indicate that modulating both blood-derived macrophages and microglia towards a pro-inflammatory phenotype may provide a powerful therapeutic approach. Both Schulz and Sevenich, and Alvaro-Espinosa et al. expand this theme further at the molecular level, in mini-reviews. Schulz and Sevenich focus specifically on tumour associated macrophages, highlighting the similarities and differences between blood-derived and brain resident populations at the molecular level. This review focuses on RNA sequencing and mass cytometry data, and the authors discuss how increasing our understanding of transcriptional and translational programs that define disease-associated macrophage functions can help us to develop macrophage-targeted therapeutics. Alvaro-Espinosa et al. review the heterogeneity that can be seen within different cellular components in the healthy and injured brain, primarily at the single cell level, and discuss how understanding the diversity of the brain microenvironment could be exploited for translational purposes. Interestingly, as described by Randrian et al., the addition of artificial intelligence tools to histological analysis of immune cell infiltration could further enhance our understanding of the TME and aid stratification of brain metastasis patients.

Brain metastasis is an increasing clinical burden, and treatments for brain metastasis are frequently ineffective. New targeted therapies, tailored to the tumour and the specialised microenvironment of the brain are needed. Corroyer-Dulmont et al. and colleagues’ mini-review evaluates the potential of radioimmunotherapy as a new approach to brain metastasis treatment. The review focuses on targeting inflammatory markers of brain metastasis for delivery of radionuclides to tumour sites. This paper also compares radioimmunotherapy with conventional whole brain radiotherapy, in terms of the balance between tumour control and healthy tissue complications. Of particular note, is the focus on treatment in the early micrometastatic stages of tumour development. Typically, brain metastases are only treated in the later stages of development, largely owing to limitations of current diagnostic imaging methods, and this is considered to be one of the main causes of ineffective treatment; treatment in the micrometastatic stages is likely to yield a much greater therapeutic response.

Imaging is frequently employed in the clinical diagnosis and management of brain metastasis. As the role of the brain’s immune response comes under greater scrutiny, it is not surprising that attention has also turned to evaluating imaging readouts of this response. Zakaria et al. provide a comprehensive review of current imaging methods and their potential for predicting and measuring the response of brain metastases to immunotherapy; these methods include structural and physiological magnetic resonance imaging (MRI) methods, as well as molecular imaging approaches. Although it is relatively early days, and study numbers can be low, several of the methods discussed have potential and, of these, molecular imaging is perhaps the most promising. For example, in a primary research study, An et al. showed a significant correlation between increased uptake of the PET tracer ^18^F-fluorodeoxyglucose (FDG) and CD68^+^ macrophages in brain metastases from primary breast cancer. Interestingly, however, increased ^18^F-FDG did not correlate with the anti-inflammatory CD163^+^ subpopulation of macrophages. Given the work by Economopoulos et al. mentioned above, the ability to selectively image pro- and anti-inflammatory macrophages in brain metastases, may be an important goal, although other PET tracers, such as ^18^F-DPA-713 (1), may provide more sensitive approaches than ^18^F-FDG. Further, as noted by Zakaria et al., amino acid PET tracers or even specifically engineered PET tracers for monitoring cell trafficking (e.g. cytotoxic T cells), likely hold greater promise for the future of brain metastasis diagnosis and monitoring, as these do not suffer from the same sensitivity limitations as ^18^F-FDG PET, owing to the high natural glucose consumption of the brain.

In recent years, one of the greatest advances in cancer therapy has been the development of therapies targeting the patient’s immune defence against cancer cells, and it is becoming clear that this may also be a promising route to effective treatment of brain metastasis. Nevertheless, these immunotherapies must be developed with the specialised microenvironment of the brain in mind. Moreover, it seems logical that the development of sensitive and specific imaging tools must go hand-in-hand with the development of novel immune-targeted therapies for the most effective treatment of brain metastasis.

## Author Contributions

All authors listed have made a substantial, direct and intellectual contribution to the work, and approved it for publication.

## Funding

This work was supported by Cancer Research UK (C5255/A15935) to NRS, The Norwegian Cancer Society (182716) to FT, and a Marie-Sklodowska Curie Action-IF (H2020-795695) to MSS.

## Conflict of Interest

The authors declare that the research was conducted in the absence of any commercial or financial relationships that could be construed as a potential conflict of interest.

## Publisher’s Note

All claims expressed in this article are solely those of the authors and do not necessarily represent those of their affiliated organizations, or those of the publisher, the editors and the reviewers. Any product that may be evaluated in this article, or claim that may be made by its manufacturer, is not guaranteed or endorsed by the publisher.
